# Mass spectrometry quantifies target engagement for a KRASG12C inhibitor in FFPE tumor tissue

**DOI:** 10.1186/s12014-023-09435-8

**Published:** 2023-10-25

**Authors:** Andrew G. Chambers, David C. Chain, Steve M. Sweet, Zifeng Song, Philip L. Martin, Matthew J. Ellis, Claire Rooney, Yeoun Jin Kim

**Affiliations:** 1 Early Oncology, AstraZeneca, One MedImmune Way, Gaithersburg, MD 20878 USA; 2 Early Oncology, AstraZeneca, Cambridge, UK

**Keywords:** Mass spectrometry, Targeted proteomics, FFPE, Translational medicine, Target engagement

## Abstract

**Background:**

Quantification of drug-target binding is critical for confirming that drugs reach their intended protein targets, understanding the mechanism of action, and interpreting dose-response relationships. For covalent inhibitors, target engagement can be inferred by free target levels before and after treatment. Targeted mass spectrometry assays offer precise protein quantification in complex biological samples and have been routinely applied in pre-clinical studies to quantify target engagement in frozen tumor tissues for oncology drug development. However, frozen tissues are often not available from clinical trials so it is critical that assays are applicable to formalin-fixed, paraffin-embedded (FFPE) tissues in order to extend mass spectrometry-based target engagement studies into clinical settings.

**Methods:**

Wild-type RAS and RASG12C was quantified in FFPE tissues by a highly optimized targeted mass spectrometry assay that couples high-field asymmetric waveform ion mobility spectrometry (FAIMS) and parallel reaction monitoring (PRM) with internal standards. In a subset of samples, technical reproducibility was evaluated by analyzing consecutive tissue sections from the same tumor block and biological variation was accessed among adjacent tumor regions in the same tissue section.

**Results:**

Wild-type RAS protein was measured in 32 clinical non-small cell lung cancer tumors (622–2525 amol/µg) as measured by FAIMS-PRM mass spectrometry. Tumors with a known KRASG12C mutation (n = 17) expressed a wide range of RASG12C mutant protein (127–2012 amol/µg). The variation in wild-type RAS and RASG12C measurements ranged 0–18% CV across consecutive tissue sections and 5–20% CV among adjacent tissue regions. Quantitative target engagement was then demonstrated in FFPE tissues from 2 xenograft models (MIA PaCa-2 and NCI-H2122) treated with a RASG12C inhibitor (AZD4625).

**Conclusions:**

This work illustrates the potential to expand mass spectrometry-based proteomics in preclinical and clinical oncology drug development through analysis of FFPE tumor biopsies.

**Supplementary Information:**

The online version contains supplementary material available at 10.1186/s12014-023-09435-8.

## Background

Kristen rat sarcoma (KRAS) protein is a membrane-associated guanosine triphosphate (GTPase) signal transducer that helps regulate cell growth, proliferation and survival. Many mutations occurring at key amino acid positions (e.g. G12, G13, and Q61) render KRAS constitutively active and drive oncogenesis in patients across a wide range of indications. Therapeutic targeting of KRAS mutants was unsuccessful for several decades due to the apparent lack of suitable drug binding pockets. However, Ostrem et al. discovered compounds that covalently bind to the cysteine in the KRASG12C mutation with high specificity and reignited the pursuit for therapeutic inhibitors [1]. The prevalence of KRASG12C mutations is highest in non-small cell lung cancer (NSCLC) patients with a prevalence of 9–14% [2, 3]. Recently, there has been widespread efforts to develop KRASG12C inhibitors as monotherapies and in combinations [4, 5].

Mass spectrometry assays offer multiplexed protein quantification in complex clinical samples (e.g. plasma, urine, and tissue) [6, 7]. These MS assays overcome several challenges of antibody-based technologies, which often suffer from non-specific binding issues. In addition, mass spectrometry assays can be developed rapidly (weeks to months) while technologies relying on monoclonal antibodies may require years for production and characterization of suitable reagents. The gold-standards for MS-based protein quantification are targeted proteomics assays, including selected reaction monitoring (SRM) and parallel reaction monitoring (PRM), with stable isotope-labeled peptides as internal standards [8]. For irreversible covalent inhibitor drugs, methods may be designed to measure the bound drug-protein complex or the free protein target. Measuring the free target protein is advantageous in early drug discovery where numerous compounds are generated, optimized, and evaluated in preclinical cellular and animal models. In this way, a single assay can be deployed to evaluate target engagement across multiple candidate compounds. In clinical samples, measuring the free target enables a single assay that evaluates target engagement and also compares the target expression levels within the studied population. Several targeted proteomics assays have been developed to quantify RASG12C protein expression in cell line models and frozen tissue samples at baseline and/or after administration of RASG12C inhibitors [9–16].

These studies have proven the utility of mass spectrometry for KRASG12C target engagement from frozen tissues; however, formalin-fixed, paraffin-embedded (FFPE) preservation is the most common storage approach for clinical tissues. Therefore, the FFPE is the standard tissue format available for both patient diagnostics and researchers developing therapeutic interventions and biomarker strategies for solid tumors. In addition, the amount of biopsied tissue samples available for clinical studies are often limited to thin sections. However, there have been a few reports of RAS mutations quantified by targeted proteomics in FFPE tissues. We previously demonstrated that a targeted data independent acquisition method enabled relative quantification of 4 KRAS mutant proteins (G12A, G12D, G12V, G13D) in FFPE tumor biopsies acquired at baseline [17]. However, this proof-of-concept work did not intend to quantify target engagement of G12C nor provide detailed assay evaluation. Hansen et al. reported an assay for FFPE tissues to evaluate a RASG12C inhibitor (ARS-1620), requiring sodium dodecyl sulphate–polyacrylamide gel electrophoresis (SDS-PAGE) to enrich for proteins in the 15–25 kDa range before in-gel trypsin digestion and PRM analysis [18]. However, methods that require SDS-PAGE are not well-suited to clinical environments due to sample low throughput, difficultly in automation, and large input sample requirements. Finally, we recently reported a multiplexed assay using FAIMS-PRM to quantify 5 proteins of clinical interest (EGFR, ESR1, HER2, KRAS, MET) in FFPE tissues, including the RASG12C mutation [19]. The spiked standard curves demonstrated excellent sensitivity for the RASG12C specific peptide spiked into formalin-fixed tissue samples. However, the study was focused on quantification of HER2 in breast cancer FFPE tissues and the clinical cohort did not include patients with a RASG12C mutation.

In this manuscript, we demonstrate a targeted proteomics mass spectrometry-based assay for precise quantification of RASG12C in preclinical and clinical FFPE tissues without a protein enrichment step (e.g. antibody pull-down or SDS-PAGE). Removing the requirement for isolating the KRAS protein before digestion and LC–MS reduces the sample input requirement for precious clinical samples and enables the panel to be expanded with additional protein biomarkers. We demonstrated the specificity of this assay by quantifying baseline RASG12C levels in FFPE samples from NSCLC patients with and without a KRASG12C mutation. We then evaluated the reproducibility for RASG12C quantification across consecutive tissue slices from the same FFPE block and among adjacent tumor regions within the same FFPE tissue section. And finally, we deployed this assay to quantify target engagement of a covalent binding RASG12C inhibitor (AZD4625) [20] in FFPE tissues from 2 xenograft models.

## Methods

### Preclinical and clinical samples

The use of animal models was approved by an internal Ethical Review Process (ERP) for the development of AZD4625 as previously described and complies with the company’s Global Bioethics Policy [10]. Xenograft models were prepared using MIA PaCa-2 (pancreatic cancer model) or NCI-H2122 (NSCLC model) and tumors were preserved as FFPE blocks. AZD4625 was synthesized as previously described [20]. In addition, a total of 32 FFPE tumor tissues from NSCLC patients were procured from commercial vendors (n = 3 from Indivumed, Frederick, MD; n = 26 from Tristar Technology Group, Washington DC, and n = 3 from Asterand Biosciences, Detroit, MI) that followed guidance for informed consent determined by separate Institutional Review Boards. The KRAS gene mutation status from these clinical tumors were supplied by the vendor.

### Tumor epithelial cell isolation

A single 10-µm-thick section was cut from the tumors removed from mice in the xenograft study. The tumor regions from clinical NSCLC tissue samples were isolated using laser microdissection as previously described [19]. Briefly, 10-µm-thick FFPE tissue sections were mounted on PEN membrane slides (Leica Microsystems, Wetzlar, Germany), deparaffinized with xylene, and stained with hematoxylin. A pathologist used an adjacent H&E-stained section to define the tumor regions for collection and analysis. Digital imaging of the H&E was conducted using the Leica Aperio AT2 scanner (Leica Microsystems, Wetzlar, Germany) at 20 × magnification. Tissue classification and image analysis was performed using Halo AI (v3.2.1851.328) software (Indica Labs, Albuquerque, New Mexico, USA). The DenseNet v2 machine learning algorithm was employed for tissue classification due to its high accuracy, versatility, and scalability. Then tumor areas were isolated by image analysis-guided laser microdissection using a LMD 6500 System (Leica Microsystems, Wetzlar, Germany) into microcentrifuge tubes.

### Sample preparation

All tissue samples (preclinical and clinical) were prepared as previously described [19]. Briefly, isolated FFPE tumor tissue was treated with RapiGest at 95 °C for 90 min to reverse formaldehyde crosslinks and denature proteins. Samples were then simultaneously reduced with Tris (2-carboxyethyl) phosphine and alkylated with chloroacetamide at 37 °C, digested with trypsin overnight, quenched with trifluoroacetic acid and desalted. Stable isotope labeled peptides (Twenty first Century Biochemicals, Marlborough, MA) matching the amino acid sequence of each endogenous target peptide were spiked at 5 fmol per 1 µg of total protein digest as measured by bicinchoninic acid assay (23,235, Thermo).

#### LC–MS

Digested samples were separated by nanoflow HPLC (Evosep One, Evosep, Odense, Denmark) and analyzed on a Orbitrap Fusion Lumos mass spectrometer with a FAIMS-PRO interface (Thermo) as previously described [19]. Briefly, a total of 1 µg of total protein digest with 5 fmol of spiked heavy peptide standards was loaded onto Evotips and separated using the 30SPD standardized method (44 min gradient). Optimized FAIMS compensation voltages were used to reduce background ions and selected ions were fragmented by higher-energy collisional dissociation and detected at 30 k resolution. Full experimental details on method development, optimized parameters, and standard curve generation have been reported [19]. The target protein concentration (in units of amol/µg) was determined by multiplying the peak area ratios (light/heavy transition) by the heavy spike (5 fmol per ug total protein).

## Results & discussion

### RASG12C assay overview

**Fig. 1:**
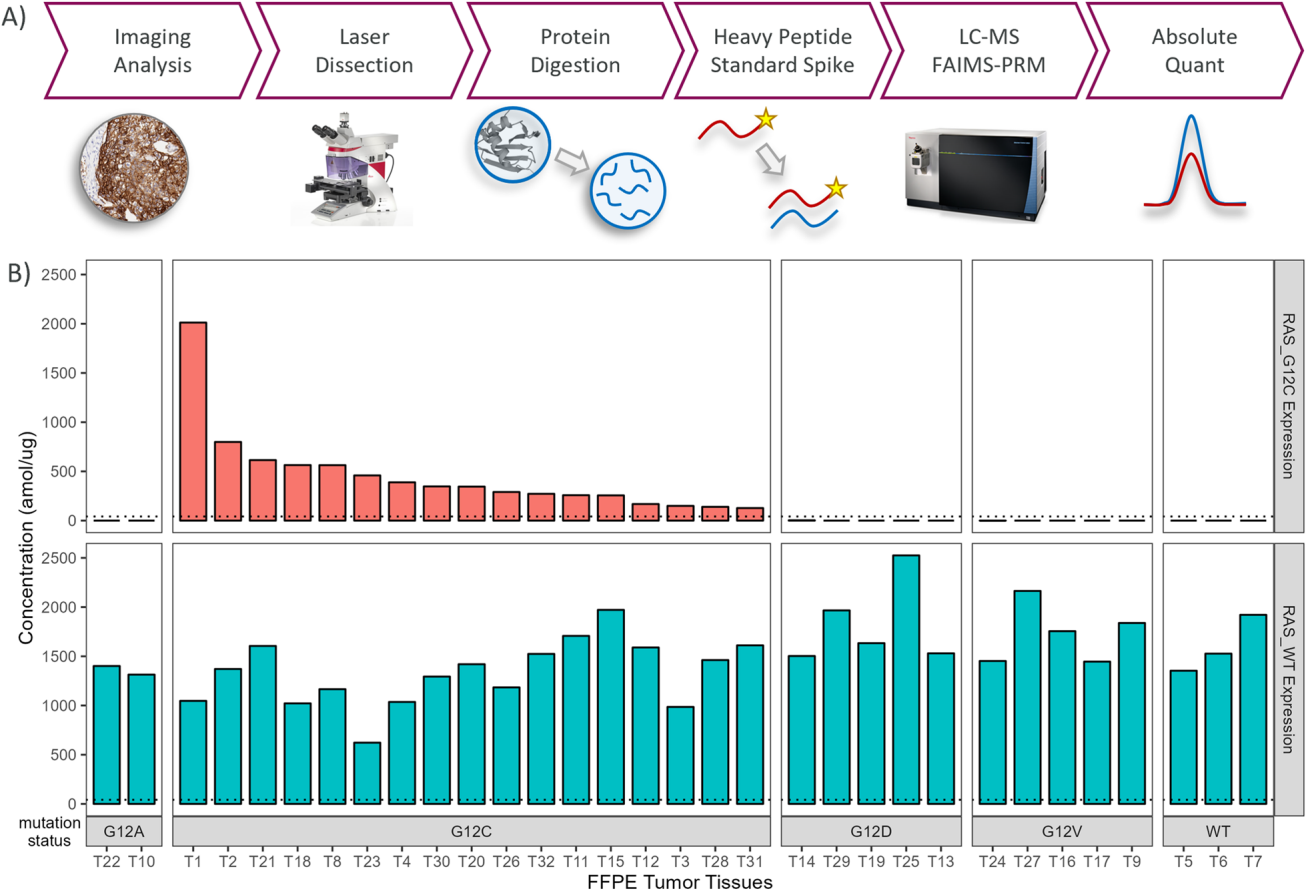
(**A**) Proteomics workflow for FFPE clinical samples analyzed by FAIMS-PRM. (**B**) The range of RASG12C and wild-type RAS protein expression in NSCLC FFPE tumor tissues (n = 32). RASG12C was only quantified above the FAIMS-PRM assay LLOQ (dashed line, 46 amol/µg) in tumors known to have the KRASG12C mutation

As illustrated in Fig. 1A, we have previously described a multiplexed FAIMS-PRM assay compatible with FFPE tissue sections that monitors both the tryptic peptides corresponding to the wild-type RAS (LVVVGA[G]GVGK) and RASG12C (LVVVGA[C]GVGK) [19]. Assay optimization included the FAIMS compensation voltage, precursor isolation width, collision energy, fragment selection and MS/MS resolution. Multiple standard curves in a formalin fixed tissue background determined the lower limit of quantification (LLOQ) of 46 amol/µg for both wild-type RAS and RASG12C. In comparison, the quantitative PRM assay from Hansen and coworkers achieved similar sensitivity (LLOQ of 5 fmol RASG12C per 100 µg tissue = 50 amol/µg); however, their assay required much larger sample inputs (100 µg total tissue protein for SDS-PAGE) [18]. For each clinical sample in our work (n = 32), laser microdissection was used to isolate the tumor region (7–21 mm^2^) from a single 10-µm-thick tissue section. These samples generated 4–21 µg of total protein digest and only 1 µg was required for FAIMS-PRM analysis. Here we now further characterize the specificity and reproducibility of our FAIMS-PRM assay in clinical FFPE samples and then quantify target engagement for a RASG12C inhibitor in xenograft FFPE tissues.

### Assay specificity

A total of 32 clinical FFPE NSCLC tumors (T1-T32) were analyzed by the FAIMS-PRM assay. This cohort includes samples with a variety of mutations at the 12th amino acid residue (G12A, G12C, G12D, G12V and WT). As shown in Fig. 1B, wild-type RAS was quantified in all tumors between 622 and 2525 amol/µg. RASG12C was only quantified in samples with known G12C mutation, demonstrating the specificity of the assay to recognize only G12C mutant protein. There was a wide range of RASG12C expression among these known KRASG12C tumors ranging 127–2012amol/µg.

### Assay reproducibility

**Fig. 2:**
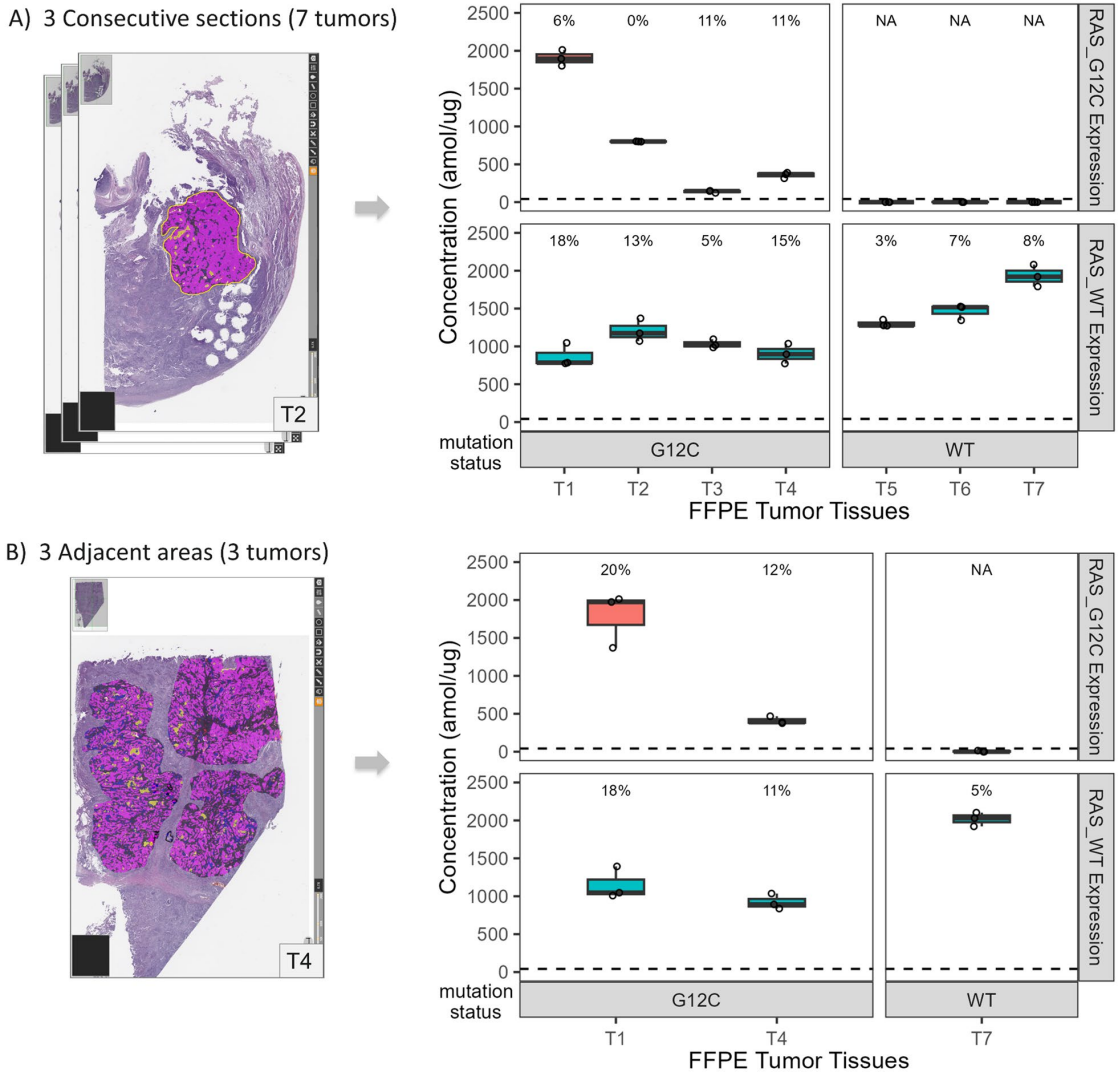
Reproducibility of the FAIMS-PRM assay for RASG12C and wild-type RAS from FFPE clinical samples. (**A**) Consecutive tumor sections, (**B**) Adjacent tumor areas. Tumors T1-T4 are known to have the KRASG12C mutation and Tumors T5-T7 are wild-type KRAS. The LLOQ for both RASG12C and wild-type RAS are 46 amol/µg, as shown by the dashed line. The %CVs for each measurement are provided above each boxplot for samples with concentrations above the LLOQ, otherwise shown as not applicable (NA)

To evaluate the reproducibility of our entire workflow in clinical samples, we cut 3 consecutive sections from NSCLC FFPE tissue samples (n = 7 tumors, T1-T4 with KRASG12C mutation; T5-T7 with wild-type KRAS) as illustrated in Fig. 2A. The same region of tumor epithelial cells was isolated from each tissue section by laser microdissection, digested with trypsin, and analyzed by FAIMS-PRM. FAIMS-PRM quantified a wide range of RASG12C protein expression in T1-T4 (146–1896 amol/µg) with high precision across the consecutive tissue Sections (6–11% CV). In addition, wild-type RAS was measured in all tumors (both wild-type KRAS and KRASG12C tumors) between 785 and 1921 amol/µg with high precision (3–18% CV). These CVs should represent the technical assay variability involved in the whole workflow (laser microdissection, digestion, microBCA, synthetic peptide spiking and LC–MS performance) as only minor biological variability is expected across the consecutive tissue sections. Overall, the RASG12C and wild-type RAS assays had low variability (≤ 11% and ≤ 18% CV, respectively) as required for biomarker studies.

To evaluate the heterogeneity of RAS expression within tumors measured by FAIMS-PRM assays, we isolated 3 adjacent tumor regions on the same slide by laser microdissection (n = 3 tumors) as shown in Additional file 1: Figures S1–S3. Figure 2B illustrates that both RASG12C and wild-type RAS were quantified with high precision across these tumor areas (5–20% CV). These CVs represent the combined error associated with the technical assay variability and the biological variability across the tissue section. Note that T4 showed slightly variation in RASG12C (20% CV) and this likely originated from biological heterogeneity.

### Application for target engagement assessment (xenograft studies)

**Fig. 3:**
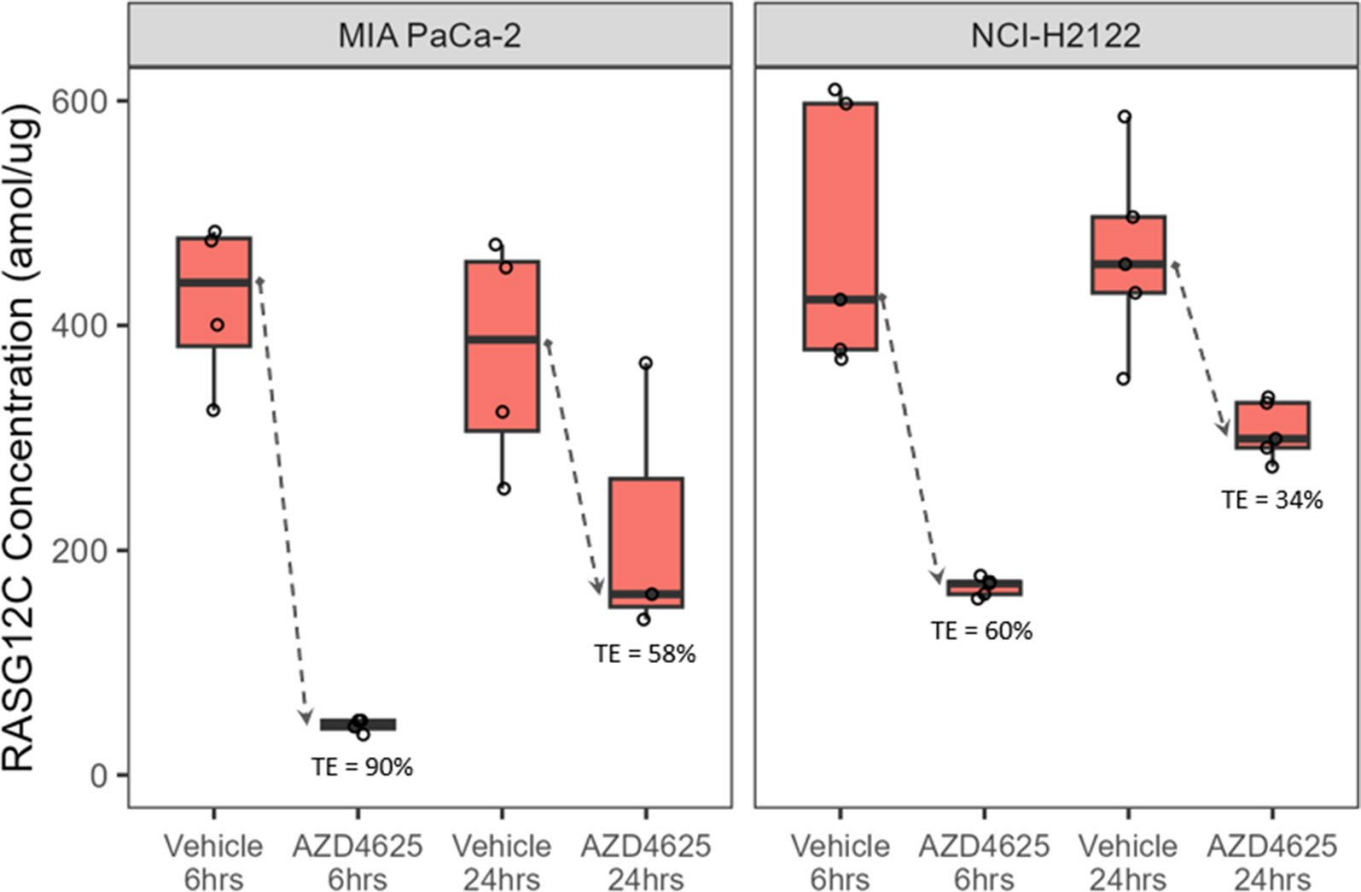
Target engagement (TE) of 100 mg per kg of AZD4625 in xenograft models (MIA PaCa-2 or NCI-H2122) is shown as the percent reduction in the median RASG12C protein compared to the time-matched vehicle control

AZD4625 is a small molecule drug candidate that binds irreversibly to RASG12C and locks the protein in its inactive GDP-bound state [20]. This orally available compound delivers high selectivity for the RASG12C mutant (sparing wild-type RAS) and potent inhibition as demonstrated in both cellular and xenograft models [10]. In this work, FAIMS-PRM was used to measure free RASG12C in FFPE tissues from 2 xenograft studies, including a pancreatic cancer model (MIA PaCa-2) and a NSCLC model (NCI-H2122). Both models harbor a KRASG12C mutation. As shown in Fig. 3, the concentrations of RASG12C in the vehicle treated animals was relatively constant across both models and between both time points (median RASG12C 387–455 amol/µg). In both studies, animals treated with 100 mg per kg AZD4625 had reductions in RASG12C. Since AZD4625 binds covalently to RASG12C, we calculated target engagement by the percent decrease in the median free RASG12C concentration when compared to the vehicle matched control. Target engagement for MIA PaCa-2 was 89.6% after 6 h and 58.4% after 24 h. Target engagement for the NCI-H2122 was 60% at 6 h and 34% at 24 h. These experiments demonstrate that targeted mass spectrometry can directly measure RASG12C protein in FFPE tissues without the need for KRAS protein enrichment (e.g. antibody pull-downs or SDS-PAGE). In this work, a single 10-µm-thick FFPE tissue section from the NCI-H2122 mouse model yielded a median value of 24 µg of total protein and only 1 µg was needed for FAIMS-PRM assay. In comparison, Hansen et al. typically used 100 µg of total protein for the SDS-PAGE to enrich for KRAS before LC–MS [18]. The lower input sample requirement for our FAIMS-PRM assay would be especially relevant for clinical samples as patient tissue biopsies are typically analyzed by multiple technologies (e.g. immunohistochemistry, DNA sequencing, RNA sequencing, LC-MS proteomics) for diagnosis, treatment selection and exploratory studies.

## Limitations

There is high sequence homology between many proteins in the RAS family, which include KRAS (Uniprot ID: P01116), HRAS (P01112), and NRAS (P01111). Therefore, all bottom-up proteomics approaches are unable to confirm that the tryptic peptide containing this mutation (LVVVGA[G]GVGK) —> (LVVVGA[C]GVGK) originated from KRAS. However, routine DNA sequencing of the tumor can confirm which RAS protein contains the mutation and then mass spectrometry can quantify the protein-level expression of RASG12C before and after therapeutic interventions. Top-down proteomics methods analyzing intact proteins by mass spectrometry may be able to differentiate mutants of RAS family members [9, 21]. However, top-down approaches require large sample inputs not suitable for most clinical samples (> 200 mg tissue was recommended from frozen tissue), require simplified protein mixtures (e.g. antibody pull-down), and are not compatible with FFPE sample formats.

## Conclusions

We have demonstrated FAIMS-PRM assay for direct quantification of free RASG12C in FFPE tissue samples without requiring protein enrichment or isolation (e.g. antibody pulldown or SDS-PAGE). This leads to significant benefits including a reduction of the sample input requirement, a streamlined workflow more amendable to high-throughput analysis, and the ability to multiplex additional protein targets. This assay was characterized by standard curves and includes internal standards in all pre-clinical and clinical samples to minimize technical variation and ensure high data quality suitable for translational medicine research. Finally, we have shown applications of this approach for assessing baseline RASG12C in patient samples and target engagement for a RASG12C inhibitor in pre-clinical models.

### Supplementary Information

**Additional file 1: Figure S1.** Three adjacent regions for clinical NSCLC tumor 1 (T1) selected for laser microdissection and analyzed by FAIMS-PRM. **Figure S2.** Three adjacent regions for clinical NSCLC tumor 4 (T4) selected for laser microdissection and analyzed by FAIMS-PRM. **Figure S3.** Three adjacent regions for clinical NSCLC tumor 7 (T7) selected for laser microdissection and analyzed by FAIMS-PRM.

## Data Availability

The datasets used and/or analyzed during the current study are available from the corresponding author on reasonable request.

## Abbreviations

FFPE: Formalin-fixed, paraffin-embedded
FAIMS: High-field asymmetric waveform ion mobility spectrometry
PRM: Parallel reaction monitoring
KRAS: Kristen rat sarcoma
GPTase: Guanosine triphosphatase
NCSLC: Non-small cell lung cancer
SRM: Selected reaction monitoring
SDS-PAGE: Sodium dodecyl sulphate–polyacrylamide gel electrophoresis
ERP: Ethical review process
LLOQ: Lower limit of quantification
WT: Wild-type
