# Supplementary material for: Mass spectrometry quantifies target engagement for a KRASG12C inhibitor in FFPE tumor tissue
Source: Clin Proteomics. 2023 Oct 25;20:47. doi: 10.1186/s12014-023-09435-8 (PMC10599008; doi:10.1186/s12014-023-09435-8)
Supplement: Supplementary file 1 — Additional file 1: Figure S1. Three adjacent regions for clinical NSCLC tumor 1 (T1) selected for laser microdissection and analyzed by FAIMS-PRM. Figure S2. Three adjacent regions for clinical NSCLC tumor 4 (T4) selected for laser microdissection and analyzed by FAIMS-PRM. Figure S3. Three adjacent regions for clinical NSCLC tumor 7 (T7) selected for laser microdissection and analyzed by FAIMS-PRM. [file 12014_2023_9435_MOESM1_ESM.pdf]

Mass Spectrometry quantifies target engagement for a KRASG12C inhibitor in FFPE tumor tissue

Andrew G. Chambers[1], David C. Chain[1], Steve M. Sweet[1], Zifeng Song[1], Philip L. Martin[1], Matthew J. Ellis[1],

Claire Rooney[2], Yeoun Jin Kim[1*]

[1]Early Oncology, AstraZeneca, Gaithersburg, MD, USA

[2]Early Oncology, AstraZeneca, Cambridge, UK

Supplementary Material

The original H&E

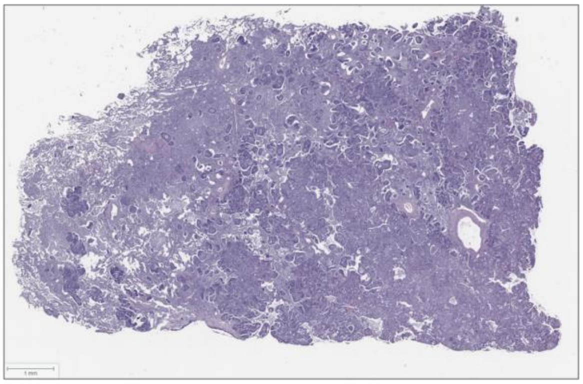

Region outlines

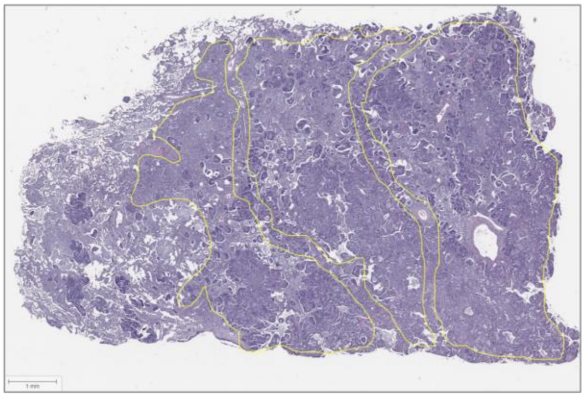

Halo AI tumor
mark up

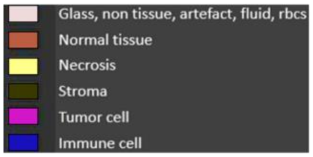

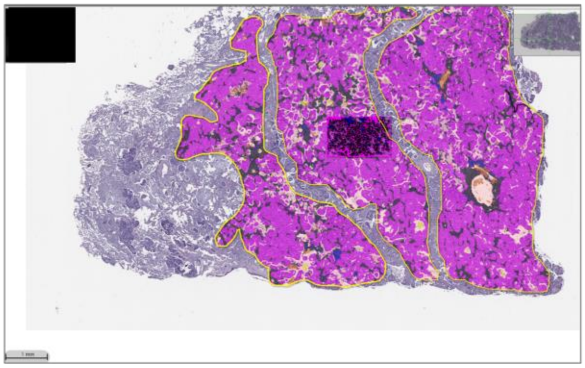

Supplemental Figure 1. Three adjacent regions for clinical NSCLC tumor 1 (T1) selected for laser microdissection and analyzed by FAIMS-PRM.

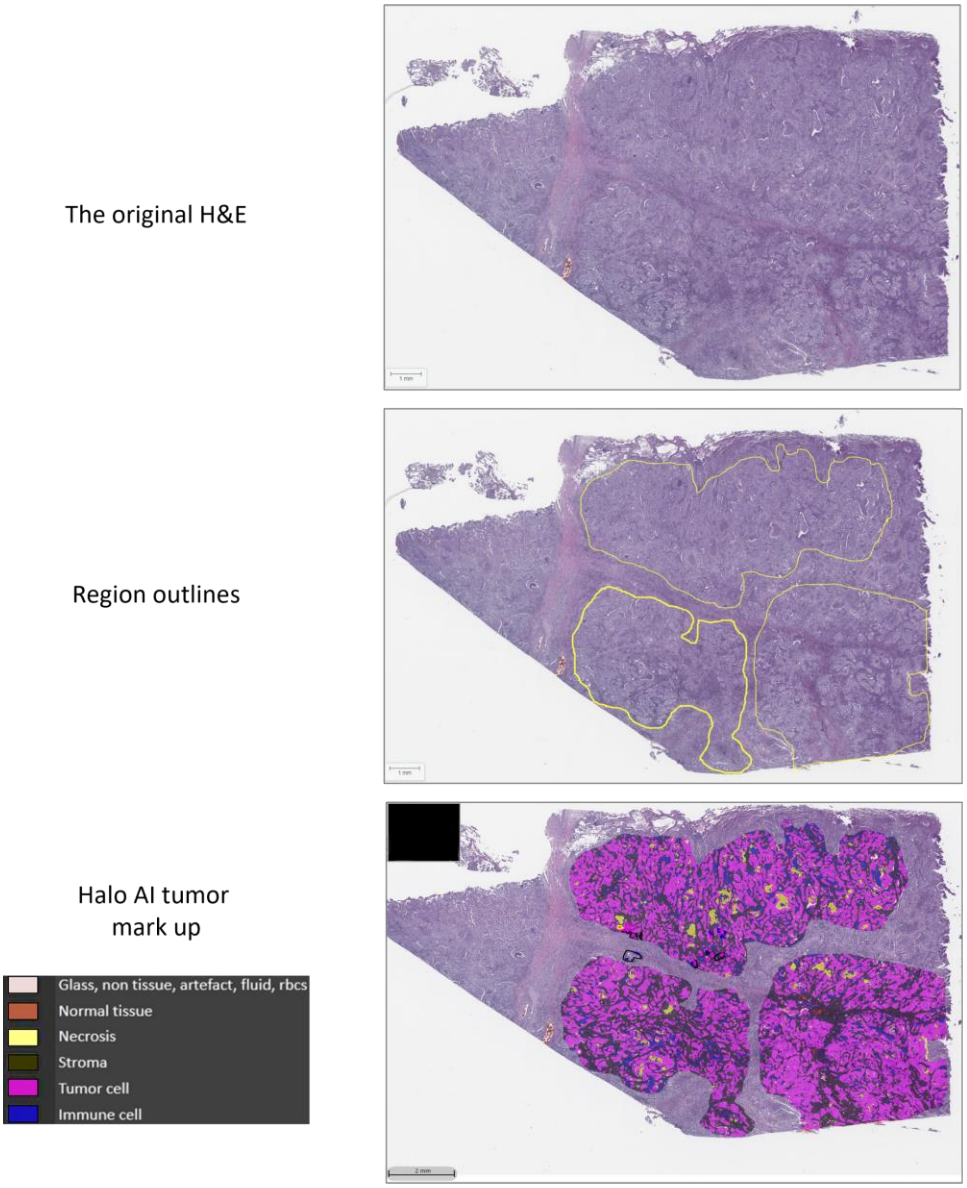

Supplemental Figure 2. Three adjacent regions for clinical NSCLC tumor 4 (T4) selected for laser microdissection and analyzed by FAIMS-PRM.

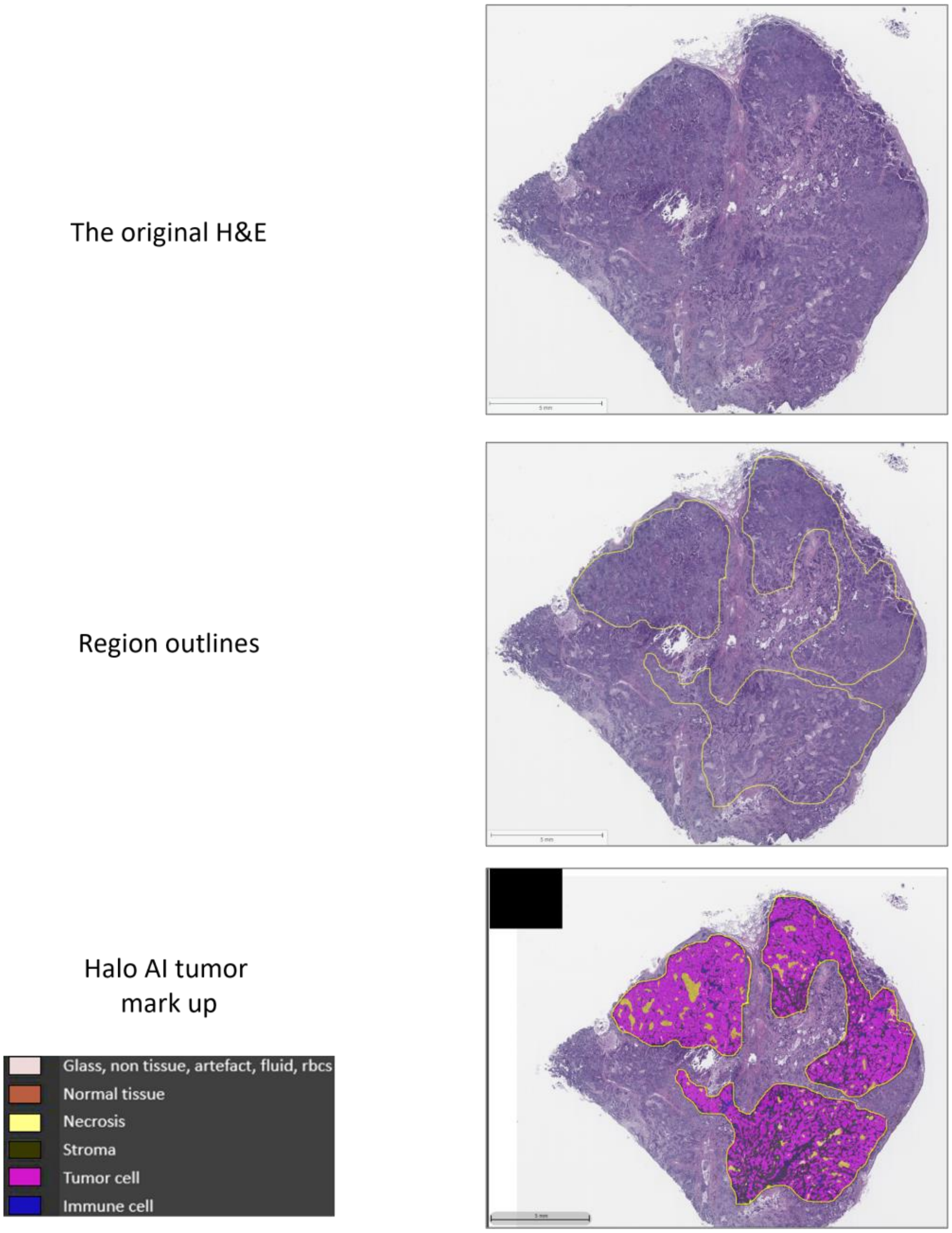

Supplemental Figure 3. Three adjacent regions for clinical NSCLC tumor 7 (T7) selected for laser microdissection and analyzed by FAIMS-PRM.
